# Myxomatous Liposarcoma of the Mediastinum: A Review of the Literature

**DOI:** 10.7759/cureus.28438

**Published:** 2022-08-26

**Authors:** Syed A Bokhari, Shahan Haseeb, Mohammad W Baig, Palwasha Qamar, Hamza H Bangash, Zahra B Manzoor, Haider Ali Babar Khan, Misbah Kaleem, Shafia Munir, Shawal Haseeb

**Affiliations:** 1 Internal Medicine, Shifa International Hospital, Islamabad, PAK; 2 Shifa Clinical Skills and Informatics Laboratory, Shifa College of Medicine, Shifa Tameer-E-Millat University, Islamabad, PAK; 3 Neurosurgery, Shifa International Hospital, Islamabad, PAK; 4 Obstetrics and Gynaecology, Hasan Medical & Surgical Complex, Hasan Abdal, PAK; 5 Obstetrics and Gynaecology, Shifa College of Medicine, Shifa Tameer-E-Millat University, Islamabad, PAK; 6 Otolaryngology, Shifa College of Medicine, Shifa Tameer-E-Millat University, Islamabad, PAK; 7 Medicine, Rawalpindi Medical University, Rawalpindi, PAK

**Keywords:** otolaryngology, radiotherapy, myxomatous liposarcoma, mediastinum, mediastinal liposarcoma

## Abstract

Myxomatous liposarcoma is an extremely rare type of mediastinal tumour that manifests in a manner comparable to other lung pathologies. Chest pain, shortness of breath, and dysphagia are the common presenting complaints. Radiological examinations or postoperative histological examinations provide the majority of the diagnostic evidence. The cornerstone of therapy consists of surgery and sometimes chemotherapy. Those who are afflicted have a better chance of experiencing favourable outcomes if they receive a diagnosis and treatment quickly.

## Introduction and background

The most frequent type of malignant soft tissue tumour is liposarcoma, and it is mesenchymal in origin [1]. It accounts for 20% of all sarcomas in the body. The retroperitoneum and the lower limbs are the most common sites where the tumour can be found. It is extremely uncommon to find liposarcoma in the mediastinum, making up less than 1% of all cases [2]. The posterior mediastinum is the part of the mediastinum that is affected by it the majority of the time. The percentage of primary mediastinal tumours that are caused by mediastinal liposarcomas ranges from 1.6% to 2.5% [3].

An increase in the MDM2 and CDK2 genes that are located on chromosome 12 is a distinguishing feature of the tumour [4]. In addition to advanced age, contact with radiation and toxic substances is also considered to be a major risk factor for the condition [5]. On the basis of histopathological characteristics, liposarcoma may be classified into the following five subtypes: well-differentiated, mucous, dedifferentiated, pleomorphic, and myxoid/round cell [6]. Well-differentiated liposarcoma is the most common subtype, whereas the second most common is myxoid liposarcoma. It constitutes 15-25% of all liposarcomas and 5% of all soft tissue tumours in the adult population [7].

This type of tumour occurs in the fourth and fifth decades of life [8]. Of individuals diagnosed with liposarcoma, 85% have symptoms, whereas the other 15% have no well-documented or distinguishing features [9]. Incidental cases are also reported [9]. The compression of the neighbouring structures by the mediastinal mass is typically what causes these symptoms, which include shortness of breath, wheezing, chest discomfort, coughing, hoarseness in the voice, and compression of the superior vena cava [10].

With this review, we aim to shed light on this relatively unknown malignancy. We want to document a comprehensive review of the literature, which will help future clinicians accurately suspect, diagnose, and manage myxoid liposarcomas.

## Review

We reviewed the existing literature using Google Scholar and PubMed. The keywords utilised in the search were myxoid and liposarcoma. These keywords yielded 22,900 results on Google Scholar and 1655 results on PubMed. We further narrowed the search to include the keywords “thorax, thoracic, mediastinal, case reports, and case series”, which gave us 7030 results on Google Scholar and 56 on PubMed. To narrow our search on Google Scholar, we used the advanced search option in which the same keywords were applied but this time limited to the title. This resulted in 10 results.

McLean et al. reported a case of myxoid liposarcoma in the anterior mediastinum in 1989 [11]. The patient was a 43-year-old female who did not have any significant symptoms. She underwent tumour resection and remained tumour free for 13 months after the surgery. Boland et al. [12] reported a case series of five patients aged 47-71 years. All patients underwent surgery for the tumour. Their report did not mention the presence of any symptoms or the size of the tumour. However, they reported varied recurrence for all patients. The details of the patients can be found in Table 1.

**Table 1:** Summary of similar cases in the literature.

Authors	Number of patients	Age	Sex	Symptoms	Treatment	Recurrence	Recurrence-free survival (months)
Schweitzer et al. [9]	1	77	Male	Neck mass, shortness of breath, hoarseness, weight loss	Surgery	None	10
McLean et al. [11]	1	43	Female	None	Surgery	None	13
Boland et al. [12]	5	76	Female	Not known	Not known	Not known	Not known
		47	Female	Not known	Surgery	None	60
		71	Female	Not known	Surgery	Residual	12
		63	Female	Not known	Surgery	Yes	24
		68	Male	Not known	Surgery	Yes	48
Hamanaka et al. [13]	1	74	Male	Dry cough	Surgery	None	8
Marulli et al. [14]	1	29	Female	Cough, dyspnea, tachycardia	Surgery	Yes	24
Plukker et al. [15]	1	5	Male	Dyspnea and chest pain	Surgery	Yes	10
Ortega et al. [16]	3	75	Male	Weight loss, dyspnea	Surgery	Yes	Not known
		66	Male	Chest pain, dysphagia	Surgery	None	Not known
		53	Female	Dysphagia	Surgery	Unknown	Not known
Chen et al. [17]	1	64	Male	Not known	Surgery	None	12
Zheng et al. [18]	1	14	Male	Chest pain, dyspnea	Surgery	None	12
Hahn et al. [19]	2	29	Male	Not known	Surgery	None	36
		20	Male	Not Known	Surgery	None	Lost to follow-up
Coulibaly et al. [20]	1	34	Female	Dyspnea	Surgery	Yes	15
Di Giammarco et al. [21]	1	62	Male	Dyspnea	Surgery	Not known	Not known
Miura et al. [22]	5	45	Female	None	Surgery	Yes	51
		62	Female	None	Surgery	Yes	28
		84	Male	None	Surgery	Yes	27
		75	Male	None	Surgery	No	3
		78	Male	Dyspnea, hoarseness	Chemotherapy	Not assessed, patient died	Not assessed, patient died
Fukai et al. [23]	1	56	Male	None	Surgery	None	36
Hirai et al. [24]	1	64	Male	Hoarseness	Surgery	No	14
Narasimman et al. [25]	1	48	Male	None	Surgery	No	12
Suster et al. [26]	2	40	Male	Enlarging mole on anterior chest, tiredness, facial swelling upon exertion	Surgery	Not known	Not known
		43	Male	Dry cough	Surgery	Yes	1

The most common symptom reported in the literature is dyspnea, but Hamanaka et al. [13] and Marulli et al. [14] reported dry cough in their patients. This symptom has not been reported in other cases. Paediatric cases have been reported as well. Plukker et al. [15] reported a case of myxoid liposarcoma in the anterior mediastinum of a five-year-old boy who presented with exertional dyspnea and chest pain. Despite undergoing surgical resection and chemotherapy, a recurrence was seen 10 months later and he died of malignancy-related complications.

Ortega et al. reported three patients with varying symptoms such as weight loss, dyspnea, and dysphagia [16]. All three patients underwent surgical resection of the tumour but the recurrence-free survival (RFS) period was not reported by them. Chen et al. [17] reported a single case of a tumour in a 64-year-old male who underwent surgery and had an RFS of 12 months.

Zheng et al. published the findings of their experience with a patient who was a 14-year-old male [18]. He was hospitalised for two weeks for chest discomfort and shortness of breath, and five days for exacerbation. Blood biochemistry revealed no unusual anomalies. CT diagnosis yielded enormous fatty neoplastic lesions in the left mediastinum (followed by neighbouring left lung local compression, collapse, and moderate left lung local inflammation), considering the differential of teratoma and liposarcoma. Following admission, a left thoracotomy and extensive thoracic tumour excision were conducted with double-lumen endotracheal intubation and intravenous anaesthesia. The lesions were consistent with myxoid liposarcoma. Because the patient showed no signs of extrathoracic or mediastinal metastases, only surgical treatment was administered, with no postoperative chemoradiotherapy. The patient was monitored for a year and no tumour recurrence was noted.

The two instances described in a case report by Hahn et al. [19] did not exhibit any recurrences or metastases; one patient with a low-grade tumour was alive with no indication of illness at 36 months; the patient who had high-grade myxoid liposarcoma was lost to follow-up. Both patients had a negative pathology report. Roughly the same findings were reported in the case reports by Coulibaly et al. [20] and Di Giammarco et al. [21] (Table 1).

An interesting presentation of mediastinal liposarcoma was reported by Schweitzer et al. [9]. The patient was a 77-year-old male who presented with a growing neck mass, difficulty buttoning his shirt, shortness of breath, hoarseness of voice, and weight loss. On examination, he had distended neck veins. Surgery was performed for the tumour. He developed respiratory difficulty post-operatively and was re-explored. At the time of publication, he had been free of disease for 10 months.

The longest RFS reported in the literature was reported by Miura et al., which was 51 months. They also reported the highest age in their case series, which was an 84-year-old female. Another notable exception in their case series was the use of the chemotherapeutic agent doxorubicin in the treatment of a 78-year-old male. All other patients cited in the literature have received surgery as initial treatment. However, doxorubicin was deemed ineffective, and two weeks later, he passed away as a result of respiratory failure. An examination of the patient's body after death indicated that the tumour was a dedifferentiated liposarcoma. However, the oesophagus, trachea, and major blood arteries had not been immediately penetrated by cancer [22].

Similar age ranges, symptoms, treatment methods, and recurrence patterns have been cited by Fukai et al. [23], Hirai et al. [24], and Narasimman et al. [25].

Myxoid liposarcoma can be distinguished from other kinds of liposarcoma by the presence of a substantial myxoid matrix. In contrast to conventional liposarcomas, which have well-differentiated areas composed of mature or normal-appearing adipocytic elements, the tumour is made up of scattered round to oval-shaped non-lipogenic small cells that are admixed with a variable number of signet-ring lipoblasts embedded in an abundant myxoid stroma. In addition, the cells are scattered throughout the abundant myxoid stroma [26]. It is a well-differentiated tumour that seldom arises in the pleural cavity as the bulk of these tumours arises in the limb, specifically the lower limb.

The clinical presentation varies from person to person. There are no clear clinical signs in the early stages. Pain, oedema, and other symptoms appear when the tumour grows in size and location and creates pressure or invasion of the surrounding tissues. Displacement of intrathoracic structures causes signs such as shortness of breath, chest discomfort, wheezing, hoarseness, superior vena cava compression, and arrhythmias, as well as heart failure.

Imaging studies such as chest radiography, CT, or magnetic resonance imaging (MRI), as well as results from histopathology, are used to diagnose liposarcoma. There is a possibility that tracheal deviation will be visible on chest radiography. It is possible to see, using CT and MRI, a fatty mass that also contains varying components of soft tissue [27].

Excision of the tumour with surgery is the recommended course of therapy for liposarcoma [28]. This enables improved exposure, which makes it possible to observe the tumour's ill-defined boundaries and distinguish the tumour from normal adipose tissue. To remove tiny mediastinal liposarcomas, in addition to open surgical procedures, minimally invasive surgery, such as video-assisted or machine-assisted thoracoscopy, can be employed. The median sternotomy or the lateral thoracotomy is the method that has the best chance of being successful and is most frequently utilised for the full removal of the mediastinal mass.

Chemotherapy and radiation are not likely to be effective in treating the tumour [29]. CDK2 inhibitors, on the other hand, can be utilised for the care of patients in cases when the mass was removed only partially.

The histological subtype of cancer has a significant role in determining the likely outcome of the disease. The least aggressive types of cancers are those that have been well-differentiated or undifferentiated. Both myxoid and pleomorphic subtypes are aggressive; they can spread to the pleural, pericardial, and diaphragmatic surfaces of the body and continue to grow. In addition to this, there is a significant rate of recurrence among these tumours [30]. Age at the time of diagnosis, size, grade, and depth of the tumour, as well as the presence or absence of tumour-free margins, are some of the factors that influence the prognosis of myxoid liposarcoma [31].

In myxoid liposarcomas, the presence of a larger fraction of the round cell component is associated with an increased risk of developing a metastatic illness. In most cases, the metastases will take place in extrapulmonary locations. The 10-year death rate rises to between 30% and 60% in cancers that have a greater proportion of round cell components [32].

Patients need to be evaluated periodically but the frequency of these evaluations should vary according to the kind of tumour present, the estimated rate of recurrence, and the degree to which the surgical excision was inadequate. Correct diagnosis helps choose the best treatment. MDM2 antibodies can aid with well-differentiated liposarcoma/atypical lipomatous tumour and dedifferentiated liposarcoma, although MDM2 amplification is suggested in unclear cases. Rarely, the hypercellular myxoid liposarcoma requires molecular testing to validate the DDIT3 gene rearrangement. Myxoid liposarcoma is an ambiguous diagnosis that may be applied arbitrarily due to a lack of a molecular signature or immunohistochemical profile. More research is needed to establish its diagnostic criteria. There are several odd or uncommon varieties that require education for correct identification.

## Conclusions

Myxomatous liposarcoma is a rare mediastinal tumour with a similar presentation to other lung pathologies. Mediastinal liposarcomas vary in morphology and can be aggressive and fatal. The diagnosis is mostly radiological or based on post-operative histology. Surgery is the mainstay of treatment. Prompt diagnosis and management can lead to favourable outcomes for those affected.
